# Sex Disparities in Resuscitation Quality Following Out of Hospital Cardiac Arrest

**DOI:** 10.1161/JAHA.123.033974

**Published:** 2024-06-27

**Authors:** Belinda Delardes, Jenna Schwarz, Tara Ralph, David Anderson, Emily Nehme, Ziad Nehme

**Affiliations:** ^1^ Center for Research and Evaluation, Ambulance Victoria Blackburn North Victoria Australia; ^2^ Department of Paramedicine Monash University Frankston Victoria Australia; ^3^ School of Public Health and Preventive Medicine Monash University St. Kilda Victoria Australia; ^4^ Department of Intensive Care and Hyperbaric Medicine The Alfred Hospital Prahran Victoria Australia

**Keywords:** emergency medical services, sex disparities, out‐of‐hospital cardiac arrest, resuscitation, Disparities, Health Equity, Social Determinants of Health, Quality and Outcomes, Cardiopulmonary Resuscitation and Emergency Cardiac Care

## Abstract

**Background:**

Women are known to be disadvantaged compared with men in the early links of the Chain of Survival, receiving fewer bystander interventions. We aimed to describe sex‐based disparities in emergency medical service resuscitation quality and processes of care for out‐of‐hospital cardiac arrest.

**Methods and Results:**

We conducted a retrospective analysis of patients who were nontraumatic with out‐of‐hospital cardiac arrest aged ≥16 years where resuscitation was attempted between March 2019 and June 2023. We investigated 18 routinely captured performance metrics and performed adjusted logistic and quantile regression analyses to assess sex‐based differences in these metrics. During the study period, 10 161 patients with out‐of‐hospital cardiac arrest met the eligibility criteria, of whom 3216 (32%) were women. There were no clinically relevant sex‐based differences observed in regard to external cardiac compressions; however, women were 34% less likely to achieve a systolic blood pressure >100 mm Hg on arrival at the hospital (adjusted odds ratio [AOR], 0.66 [95% CI, 0.47–0.92]). Furthermore, women had a longer time to 12‐lead ECG acquisition after return of spontaneous circulation (median adjusted difference, 1.00 minute [95% CI, 0.38–1.62]) and 33% reduced odds of being transported to a 24‐hour percutaneous coronary intervention‐capable facility (AOR, 0.67 [95% CI, 0.49–0.91]). Resuscitation was also terminated sooner for women compared with men (median adjusted difference, −4.82 minutes [95% CI, −6.77 to −2.87]).

**Conclusions:**

Although external cardiac compression quality did not vary by sex, significant sex‐based disparities were seen in emergency medical services processes of care following out‐of‐hospital cardiac arrest. Further investigation is required to elucidate the underlying causes of these differences and examine their influence on patient outcomes.

**Nonstandard Abbreviations and Acronyms**
ECC: external cardiac compression
OHCA: out‐of‐hospital cardiac arrest
VACAR: Victorian Ambulance Cardiac Arrest Registry

**Clinical Perspective**

**What Is New?**

- No clinically relevant sex‐based differences were seen in the quality of external cardiac compressions delivered by emergency medical services providers.
- Women who achieved prehospital return of spontaneous circulation were less likely to achieve a systolic blood pressure >100 mm Hg on arrival at the emergency department, have longer time to 12‐lead ECG acquisition, and were less likely to be taken to a percutaneous coronary intervention‐capable facility.
- Where return of spontaneous circulation was not achieved prehospitally, women received shorter resuscitation attempts compared with men.

**What Are the Clinical Implications?**

- Postresuscitation care should be improved for women, although it is not currently known how best to address this sex gap.
- Ensuring that women receive an adequate duration of resuscitation before prehospital termination may increase the number of women who survive out‐of‐hospital cardiac arrest.

Surviving an out‐of‐hospital cardiac arrest (OHCA) relies on efficient and effective care at all stages of the Chain of Survival.^1^ Women have been shown to be disadvantaged compared with men in the early links of the Chain of Survival. Women are more likely to have an unwitnessed OHCA, delaying the vital initiation of the resuscitation chain.^2^, ^3^ Furthermore, following recognition of OHCA, women are less likely to receive bystander cardiopulmonary resuscitation (CPR) and public access defibrillator application and defibrillation.^4^, ^5^, ^6^, ^7^, ^8^

Despite these disadvantages, a recent systematic review and meta‐analysis found no sex‐based differences in the adjusted rate of survival to hospital discharge or at 30 days after OHCA.^9^ Importantly, this review found substantial heterogeneity on sex‐based differences in OHCA survival, and the authors suggested that further investigation of resuscitation factors that are not reported in the Utstein template is necessary to elucidate latent inequalities in patient care.

There is evidence that suggests that women are less likely to receive targeted postresuscitation care and are more likely to have active resuscitation care withdrawn prematurely.^9^ Exploring sex differences in the application of care in the prehospital setting is an important step toward improving patient‐centered care outcomes at all stages of the Chain of Survival.^1^, ^10^ Emergency medical services (EMS) providers contribute to postresuscitation care through their immediate management before hospital handover, as well as through their decision whether to bypass local hospitals and transport to a tertiary cardiac center. Direct transport of the patient to a tertiary cardiac center can increase patients' access to postresuscitation care interventions and has been associated with increased survival.^11^, ^12^, ^13^

Previously, obtaining objective data about the quality of EMS resuscitation has been limited to retrospectively written patient care records; however, recent technological advances allow increased opportunity to use objective data collected in real time. Although previous studies have found EMS providers are less likely to begin resuscitation on women compared with men,^14^ and women are less likely to receive intravenous/intraosseous access and subsequent adrenaline or antiarrhythmic agents,^15^ there is no existing evidence related to sex‐based differences in external cardiac compression (ECC) quality. The development of CPR feedback pads, which provide both live and recorded audiovisual feedback on the quality of ECC, allows insights into the quality of the fourth link in the Chain of Survival, early advanced life support.

The aim of this study was to examine sex‐based disparities in EMS resuscitation quality and processes of care for OHCA.

## METHODS

### Data Availability

The data that support the findings of this study are available upon reasonable request by emailing ziad.nehme@ambulance.vic.gov.au.

### Study Design

A retrospective analysis of cases from the VACAR (Victorian Ambulance Cardiac Arrest Registry) was undertaken for all OHCA cases where resuscitation was attempted between March 2019 and June 2023. Patients were excluded if sex was not recorded, their age was <16 years, they had an advance care directive stipulating do not resuscitate, or the OHCA was precipitated by a traumatic event or was EMS witnessed. This project received ethical approval from Monash University Human Research Ethics Committee (project ID: 21046). Implied consent was gained from patients, with the institutional privacy policy available online.^16^

### Setting

Ambulance Victoria is the single state‐wide provider of EMS in the state of Victoria, Australia. The EMS provider services a population of 6.5 million people across 227 000 km^2^. Access to EMS is activated through a single nationwide telephone number (000). Suspected cardiac arrests receive telephone CPR instructions for bystanders^17^ and a simultaneously activated 3‐tiered response consisting of basic life support first responders (metropolitan Melbourne and regional centers), advanced life support paramedics, and intensive care paramedics.^18^ Ambulance Victoria has also integrated the alerting of smartphone‐activated volunteer responders (GoodSAM) into their emergency dispatch system for eligible cases, which involves responding first aid‐trained volunteers to OHCAs to provide initial resuscitation while awaiting EMS, as described elsewhere.^19^

Ambulance Victoria cardiac arrest treatment guidelines are outlined in publicly accessible clinical practice guidelines and are divided into medical and traumatic protocols.^20^ Paramedics are instructed to use a high‐performance CPR approach for medical cardiac arrests, prioritizing rapid defibrillation and minimizing interruptions to ECC.^21^ Paramedics use feedback pads that record the chest compression depth, rate, recoil velocity, as well as the overall chest compression fraction, single longest pause, pre‐ and postshock pauses, 12‐lead electrocardiography capture, and end‐tidal CO_2_ capnography. This recording is then uploaded to a central online database.

The EMS employs dedicated resuscitation coordinators who audit paramedic performance of all OHCAs where a resuscitation attempt was provided by EMS to ascertain resuscitation performance metrics using patient care records and the defibrillator recording.

### Data Source

Data were sourced from the VACAR, which is a population‐based clinical quality registry of all OHCA attended to by EMS in Victoria, as previously described.^22^ The investigators had full access to the VACAR. Sex is recorded by the treating paramedics, who have a drop‐down selection of male, female, or other/unknown. Current data capture therefore does not adequately capture patients whose sex falls outside of the sex binary.

Performance data are divided into the 5 domains of early recognition, quality ECC, defibrillation, advanced interventions, and postresuscitation care,^23^ aligning with the Chain of Survival.

### Outcomes

A total of 18 metrics across the 5 performance data domains were measured, including a mix of binary, proportional, and time‐based performance metrics, as outlined in Table 1.

**Table 1:** Resuscitation Performance Metric Definitions as Per the Victorian Ambulance Cardiac Arrest Registry

Domain	Metric	Measurement unit	Definition	Data source
Early recognition	Time to place pads	Minutes	Time from arrival at patient until defibrillation pads connected to patient	Patient care record; defibrillator recording
	Compressions during pad placement	Yes/no	Recorded yes if ECC waveform is present in pad impedance display	Defibrillator recording
	Correct initial rhythm identification	Yes/no	Recorded yes if 2 independent reviewers from the Victorian Ambulance Cardiac Arrest Registry agree with the paramedics' decision to defibrillate (or disarm) during the initial rhythm analysis	Defibrillator recording
ECC quality	Mean compression rate	Compressions per minute	Mean rate of compressions per minute	Defibrillator recording
	Mean compression depth	Centimeters	Mean depth of compressions	Defibrillator recording
	Chest compressions fraction	Percentage	Proportion of resuscitation attempt where compressions were occurring	Defibrillator recording
	Average recoil velocity	Millimeters per second	The mean manual release velocity during compressions	Defibrillator recording
Defibrillation	Time to first defibrillation	Minutes	Time between arrival at patient and first defibrillation	Defibrillator recording
	Average preshock pause	Seconds	Mean length of time between hands off chest and delivery of defibrillation	Defibrillator recording
	Average postshock pause	Seconds	Mean length of time between defibrillation and hands off chest postdefibrillation	Defibrillator recording
Advanced interventions	Time to insert supraglottic airway	Minutes	Time between arrival at patient and insertion of supraglottic airway	Patient care record
	Intubation first pass rate	Yes/no	Recorded yes if patient care record indicates patient was intubated successfully on first pass, as indicated by end‐tidal CO_2_ capnography waveform	Patient care record
	Time to administer first bolus adrenaline	Minutes	Time between arrival of fourth personnel at patient and administration of initial bolus of adrenaline	Patient care record
	Time to administer first bolus amiodarone	Minutes	Time between arrival of fourth personnel at patient or third defibrillation (whichever is later) and administration of initial bolus of amiodarone	Patient care record
Postresuscitation care	Systolic blood pressure ≥100 mm Hg on arrival to hospital	Yes/no	Patient has a reported systolic blood pressure ≥100 mm Hg on arrival at emergency department	Patient care record
	Time to 12‐lead ECG acquisition	Minutes	Time between sustained ROSC (≥10 min) and time of first 12‐lead ECG after ROSC	Defibrillator recording
	Transport to 24‐hour PCI facility	Yes/no	Recorded yes if the patient is transported to a 24‐hour PCI‐capable hospital if the initial rhythm is shockable or the presumed cause is cardiac, and the patient is transported with ROSC or mechanical ECC	Patient care record
	Resuscitation duration	Minutes	Duration of ECC delivered by paramedics where resuscitation is terminated prehospitally	Patient care record; defibrillator recording

ECC indicates external cardiac compressions; PCI, percutaneous coronary intervention; and ROSC, return of spontaneous circulation.

### Statistical Analysis

Statistical analyses were undertaken using Stata Statistical Software 18 (StataCorp, College Station, TX). Baseline characteristics are presented as frequencies and proportions for categorical data and medians and interquartile ranges for continuous data.

Categorical outcomes were assessed with multivariable logistic regression, and continuous variables using quantile regression. All models were adjusted for patient age, arrest cause (presumed cardiac versus other nontraumatic cause), and location of the OHCA (private residence, aged care facility, public location, or other including medical clinics, corrections facilities). Results of the logistic regression analyses are presented as adjusted odds ratio (AOR) and 95% CI. Results from quantile regression are presented as median adjusted difference and 95% CI. Variables with statistically significant differences are visually represented using kernel density plots comparing women and men. Sensitivity analyses were performed, which also included initial presenting rhythm, witnessed status, and presence of bystander CPR on paramedic arrival as confounders.

Missing data were handled using pairwise deletion.

## RESULTS

### Baseline Characteristics

**Figure 1:**
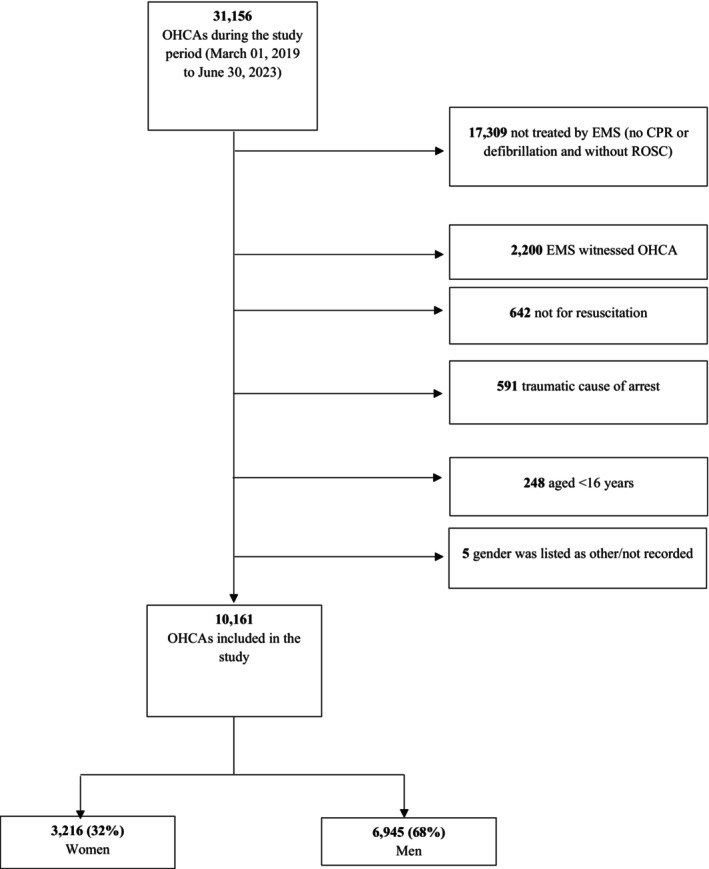
Patient selection for OHCAs occurring between March 2019 and June 2023. CPR indicates cardiopulmonary resuscitation; EMS, emergency medical services; OHCA, out‐of‐hospital cardiac arrest; and ROSC, return of spontaneous circulation.

We included 10 161 OHCA cases, the majority (68%) of which were men (Figure 1). The baseline characteristics for women were less favorable, as shown in Table 2. On average, women were older and were more likely to present in a nonshockable rhythm. Women were also less likely to have an OHCA witnessed by a bystander, receive bystander CPR, or arrest in a public location.

**Table 2:** Characteristics of Patients With OHCA With an Attempted Resuscitation Between March 2019 and June 2023

Characteristic	All included patients (n=10 161)	Women (n=3216)	Men (n=6945)
Ambulance response time, min, median (IQR)	9.3 (7.4–12.1)	9.3 (7.5–12.2)	9.3 (7.3–12.0)
Missing, n (%)	7 (0.1%)	0 (0.0%)	7 (0.1%)
Age, y, median (IQR)	68 (53–79)	69 (53–81)	67 (53–78)
Missing, n (%)	12 (0.1%)	4 (0.1%)	8 (0.1%)
Location of OHCA, n (%)			
Private residence	7550 (74.3%)	2571 (79.9%)	4979 (71.7%)
Public location	1365 (13.4%)	209 (6.5%)	1156 (16.7%)
Medical facility including aged care facilities	667 (6.6%)	285 (8.9%)	382 (5.5%)
Other	566 (5.6%)	144 (4.5%)	422 (6.1%)
Missing	13 (0.1%)	7 (0.2%)	6 (0.1%)
OHCA witnessed by bystander, n (%)	5110 (50.3%)	1526 (47.4%)	3584 (51.6%)
Received bystander CPR, n (%)	7483 (73.6%)	2323 (72.2%)	5160 (74.3%)
Presumed cardiac cause, n (%)	8121 (79.9%)	2493 (77.5%)	5628 (81.0%)
Initially shockable rhythm, n (%)	2699 (26.6%)	509 (15.8%)	2190 (31.5%)
Missing	35 (0.3%)	14 (0.4%)	21 (0.3%)
Any ROSC, n (%)	3574 (35.2%)	1070 (33.3%)	2504 (36.1%)
Missing	0 (0.0%)	0 (0.0%)	0 (0.0%)
Event survival, n (%)	2953 (29.1%)	885 (27.5%)	2068 (29.8%)
Missing	7 (0.1%)	1 (0.0%)	6 (0.1%)
Survival to hospital discharge, n (%)	1.025 (10.2%)	219 (6.9%)	806 (11.7%)
Missing	103 (1.0%)	30 (0.9%)	73 (1.1%)

CPR indicates cardiopulmonary resuscitation; IQR, interquartile range; OHCA, out‐of‐hospital cardiac arrest; and ROSC, return of spontaneous circulation.

**Figure 2:**
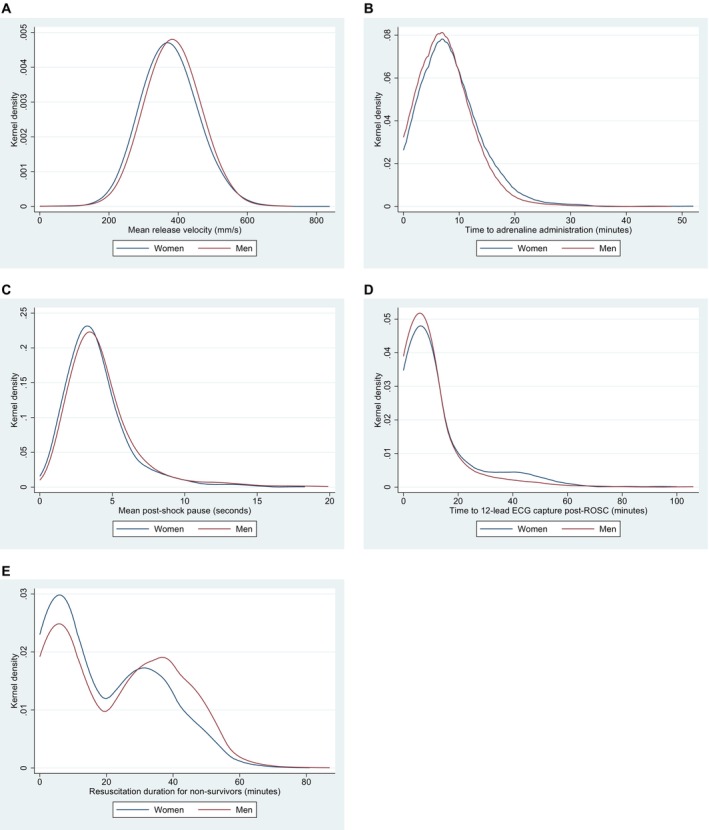
Kernel density estimation plots for unadjusted outcomes with significant sex‐based differences, by sex, between March 2019 and June 2023. **A**, The mean manual release velocity during compressions. **B**, Time between arrival of fourth personnel at patient and administration of initial bolus of adrenaline. **C**, Mean length of time between defibrillation and hands off chest postdefibrillation. **D**, Time between sustained ROSC (≥10 minutes) and time of first 12‐lead ECG after ROSC. **E**, Duration of ECC delivered by paramedics where resuscitation is terminated prehospitally. ECC indicates external cardiac compressions; and ROSC, return of spontaneous circulation.

Resuscitation performance metrics are shown in Table 3, and outcomes with significant sex‐based differences are displayed graphically in Figure 2A through 2E.

**Table 3:** Resuscitation Performance and Outcomes of Patients With Out‐of‐Hospital Cardiac Arrest With an Attempted Resuscitation Between March 2019 and June 2023

Variable		N (total patients =10 161)	All included patients	Women (n=3216)	Men (n=6945)	Adjusted odds ratio (95% CI)	Adjusted median difference (95% CI)	Missing from regression analysis, n (%)
Recognition	Time, min, to place pads, median (IQR)	4950	1 (1 to 2)	1 (1 to 2)	1 (1 to 2)	…	0.00 (0.00 to 0.00)	12 (0.2%)
	Compressions occurring during pad placement, n (%)	4896	4505 (92.0%)	1359 (91.9%)	3146 (92.0%)	0.99 (0.79 to 1.25)	…	12 (0.2%)
	Correct initial rhythm identification, n (%)	5008	4657 (93.0%)	1400 (93.1%)	3257 (93.0%)	1.04 (0.82 to 1.32)	…	13 (0.3%)
ECC quality	Mean compression rate, median (IQR)	6565	115 (110 to 120)	115 (110 to 120)	115 (110 to 120)	…	0.41 (−0.06 to 0.89)	13 (0.2%)
	Mean compression depth, median (IQR)	6570	5.8 (5.1 to 6.5)	5.8 (5.1 to 6.4)	5.8 (5.1 to 6.5)	…	0.05 (−0.01 to 0.11)	13 (0.2%)
	Chest compressions fraction, median (IQR)	6617	92 (89 to 93)	92 (88 to 93)	91 (89 to 93)	…	0.05 (−0.01 to 0.12)	13 (0.2%)
	Average recoil velocity, mm/s, median (IQR)	6568	379 (337 to 424)	371 (329 to 417)	383 (341 to 427)	…	−9.13 (−13.24 to −5.01)	13 (0.2%)
Defibrillation	Time, min, to first defibrillation, median (IQR)	1552	2 (1 to 3)	2 (1 to 3)	2 (1 to 3)	…	0.00 (−0.30 to 0.30)	1 (0.1%)
	Average preshock pause, s, median (IQR)	2979	5.7 (4.0 to 8.3)	6.0 (3.9 to 9.0)	5.6 (4.0 to 8.2)	…	0.20 (−0.12 to 0.52)	3 (0.1%)
	Average postshock pause, s, median (IQR)	2979	3.6 (2.8 to 4.8)	3.4 (2.7 to 4.5)	3.6 (2.8 to 4.9)	…	−0.24 (−0.40 to −0.07)	3 (0.1%)
Advanced interventions	Time, min, to insert supraglottic airway, median (IQR)	4505	3 (2 to 4)	3 (2 to 4)	3 (2 to 4)	…	0.00 (−0.05 to 0.05)	9 (0.2%)
	Intubation first pass rate, n (%)	3283	2748 (83.7%)	786 (84.8%)	1962 (83.2%)	1.09 (0.88 to 1.34)	…	7 (0.2%)
	Time, min, to administer first bolus adrenaline, median (IQR)	3662	7 (4 to 10)	8 (5 to 11)	7 (4 to 10)	…	1.00 (0.53 to 1.47)	9 (0.2%)
	Time, min, to administer first bolus amiodarone, median (IQR)	1015	2 (1 to 5)	2 (1 to 6)	2 (1 to 5)	…	0.00 (−0.84 to 0.84)	0 (0.0%)
Postresuscitation care	Systolic blood pressure ≥100 mm Hg on arrival at the hospital, n (%)	1744	1572 (90.3%)	458 (87.2%)	1116 (91.6%)	0.66 (0.47 to 0.92)	…	2 (0.1%)
	Time, min, to 12‐lead ECG acquisition, median (IQR)	1740	6 (4 to 11)	6 (4 to 14)	5 (4 to 10)	…	1.00 (0.38 to 1.62)	3 (0.2%)
	Transport to 24‐hour PCI facility, n (%)	2478	2.262 (91.3%)	593 (88.5%)	1669 (92.3%)	0.67 (0.49 to 0.91)	…	11 (0.4%)
	Resuscitation duration for nonsurvivors, min, median (IQR)	7035	20 (5 to 35)	14 (5 to 32)	24 (6 to 37)	…	−4.82 (−6.77 to −2.87)	21 (0.3%)

ECC indicates external cardiac compressions; IQR, interquartile range; and PCI, percutaneous coronary intervention.

### Early Recognition

The median time from arrival at patient to placement of defibrillation pads was 1 minute for both women and men, with no sex‐based difference following adjustment. Likewise, compressions occurred during pad placement for 92% of patients, with women having similar odds to men following adjustment (AOR, 0.99 [95% CI, 0.79–1.25]). Accurate rhythm recognition occurred in 93% of patients, with no difference between women and men (AOR, 1.04 [95% CI, 0.82–1.32]).

### ECC Quality

Overall, the median compression rate was 115 compressions per minute, with no differences between women and men (median adjusted difference, 0.50 compressions per minute [95% CI, −0.01 to 1.01]). The median compressions depth also showed no difference between women and men (median adjusted difference, 0.05 cm [95% CI, −0.01 to 0.11]). The chest compression fraction was slightly higher for women compared with men (92% versus 91%); however, the adjusted difference was nonsignificant (median adjusted difference, 0.08% [95% CI, −0.17 to 0.33]). Median chest recoil velocity was slower for women compared with men (371 mm/s versus 383 mm/s), and this remained significant following adjustment (median adjusted difference, −9.13 mm/s [95% CI, −13.24 to −5.01]).

### Defibrillation

For patients receiving defibrillation, the median time to first defibrillation was 2 minutes, whereas the median preshock pause was 5.7 seconds. There were no sex‐based adjusted differences for either outcome (time to defibrillation: median adjusted difference, 0.00 minutes [95% CI, −0.30 to 0.30]; preshock pause: median adjusted difference, 0.20 seconds [95% CI, −0.12 to 0.52]). In contrast, women received a shorter postshock pause compared with men (3.4 versus 3.6 seconds; median adjusted difference, −0.24 seconds [95% CI, −0.40 to −0.07]).

### Advanced Interventions

The median time from paramedic arrival to insertion of a supraglottic airway device was 3 minutes overall, and first‐pass intubation was achieved in 83.7% of the overall cohort. There were no sex‐based differences for either outcome (time to supraglottic airway: median adjusted difference, 0.00 minutes [95% CI, −0.05 to 0.05]; first pass intubation success: AOR, 1.09 [95% CI, 0.88–1.34]). In contrast, time to adrenaline administration was longer for women compared with men (8 minutes versus 7 minutes; median adjusted difference, 1.00 minute [95% CI, 0.53–1.47]).

### Postresuscitation Care

Postresuscitation care was inferior for women within all performance metrics. Women had reduced odds of achieving a systolic blood pressure >100 mm Hg on arrival at the hospital (87.2% versus 91.6%), which remained significant after adjustment (AOR, 0.66 [95% CI, 0.47–0.92]). The median time to 12‐lead ECG after return of spontaneous circulation was 6 minutes for women compared with 5 minutes for men (median adjusted difference, 1.00 minute [95% CI, 0.38–1.62]). This translated to a higher proportion of women receiving a 12‐lead ECG after loading while en route to a hospital (11.5% versus 6.7%; AOR, 0.60 [95% CI, 0.40–0.89]).

Similarly, women were less likely to be transported to a 24‐hour percutaneous coronary intervention (PCI)‐capable facility than men (88.5% versus 92.3%), and this remained significant after adjustment (AOR, 0.67 [95% CI, 0.49–0.91]). Lastly, in patients who died on scene, resuscitation was terminated at a median time of 14 minutes for women compared with 24 minutes for men, and this difference was significant after adjustment (median adjusted difference, −4.82 minutes [95% CI, −6.77 to −2.87]). As shown in Figure 2E, there was a bimodal distribution of resuscitation duration for nonsurvivors, with the major peak representing paramedic decision to withhold resuscitation in the initial stages of the resuscitation attempt and the minor peak representing ceased resuscitations following the recommended minimum duration as per the internal guideline. All results were consistent in our sensitivity analyses (Table S1), with the exception of resuscitation duration for patients who died at scene, in which the adjusted median difference remained significant but reduced to −1.05 (95% CI, −2.09 to 0.00).

## DISCUSSION

Our analysis of resuscitation quality metrics among >10 000 patients with OHCA highlights several sex‐based disparities in resuscitation care. Compared with men, women experienced longer delays in adrenaline administration and 12‐lead ECG acquisition. Women were also less likely to receive perfusion management resulting in a systolic blood pressure >100 mm Hg on arrival to hospital or be transported to a 24‐hour PCI‐capable facility. In nonsurvivors, women also received shorter resuscitation attempts compared with men. The only metric in which women received superior care to men was postshock pauses.

Perman et al investigated public perceptions of why women receive less bystander CPR compared with men following OHCA, and found a common misconception that women are weak and frail and therefore more prone to injury.^24^ Our study found no sex‐based differences in the rate or depth of compressions, suggesting this misconception is likely not perpetuated by EMS providers. Despite this, recoil velocity in our study was slower for women compared with men (median difference, −9.13 mm/s [95% CI, −13.24 to −5.01]). This finding mimics previous research that suggests that, despite similar chest compression depth, structural differences in the elasticity of the female chest wall may be responsible for a slower recoil velocity compared with male patients.^25^, ^26^

The majority of sex‐based disparities in resuscitation quality and processes of care seen in our study relate to the postresuscitation care domain. This is in line with previous research showing that women are less likely to receive invasive postresuscitation care therapies such as urgent coronary angiography and PCI.^27^, ^28^, ^29^ Identifying ischemic changes on a 12‐lead ECG informs the EMS’ decision to transport to a PCI‐capable facility, as well as activate code ST‐segment–elevation myocardial infarction protocols that have been associated with reduced door‐to‐balloon time and mortality rates.^30^ Previous literature in our region reported similar rates of ST‐segment–elevation myocardial infarction among men and women who presented postresuscitation for an initially shockable rhythm.^31^ Despite this, women in our study had a delay to 12‐lead ECG acquisition compared with men, suggesting that investigating a cardiac cause of OHCA may be less prioritized for women. Although the median difference was only 1 minute longer for women compared with men, women in our study were more likely (11.5% versus 6.7%) to have their 12‐lead ECG taken while en route to a hospital rather than on scene. The acquisition of a 12‐lead ECG on scene allows paramedics to appropriately direct the patient to a PCI‐capable facility and give adequate notification to the receiving hospital. This finding may have contributed to the sex‐based disparity in PCI‐facility transports.

Furthermore, among patients with a presumed cardiac cause, women were 33% less likely to be transported to a 24‐hour PCI‐capable facility, even after adjustment for OHCA baseline characteristics. There is current contention on the importance of receiving postresuscitation care in a tertiary cardiac center. Although a recent systematic review suggested that receiving postresuscitation care at a tertiary cardiac facility improves patient outcomes,^13^ a prospective, multicenter, randomized clinical trial in London, United Kingdom published shortly after showed no difference in outcomes.^32^ Locally in the Victorian region, direct transfer to a PCI‐capable center was associated with a 40% increase in survival to hospital discharge.^33^ Inequitable access to tertiary cardiac care centers for men and women following return of spontaneous circulation as a result of EMS decision making may suggest an implicit bias from EMS that women are less likely to require tertiary cardiac care.

Women in our study were less likely to have a systolic blood pressure of at least 100 mm Hg on arrival at the hospital, although we were not able to establish how pharmacologically aggressive patients were managed by paramedics. Patients who remain severely hemodynamically compromised despite aggressive perfusion therapy may be more likely to be diverted to the closest hospital in lieu of further travel for a PCI‐capable facility. Some goal‐directed therapies within our system are only available after 12‐lead ECG acquisition, such as anticoagulants and thrombolysis administration. Women in our study were less likely to have a 12‐lead ECG acquired before the transport decision and thus had a reduced opportunity to receive goal‐directed therapies that may have stabilized their hemodynamic status to allow for longer transport to a PCI‐capable facility. These considerations are already stated in the *Clinical Practice Guideline* for return of spontaneous circulation management in Victoria^20^; however, further education on the topic may be necessary to elicit cultural change.

Consistent with previous studies, women in our study were older and more likely to arrest in a residential aged care facility, while being less likely to arrest in public, have a witnessed OHCA, receive bystander CPR, or present in a shockable rhythm compared with men. For patients whose resuscitation was terminated by EMS on scene, women in our study received shorter resuscitation attempts compared with men. However, it is noteworthy that inclusion of the baseline characteristics in our multivariable model reduced the median difference in resuscitation duration to only 1 minute.

### Limitations

In this study, we were reliant on the paramedics' assumption of the patient's sex as opposed to the patient's stated sex, which may differ. However, our study focused on paramedic behaviors in response to the patient sex, and the findings are therefore relevant to patients who paramedics perceive as women, even if they self‐identify otherwise. Some variables are derived from the paramedic‐written patient care records, and inaccuracies may arise from the retrospective recording of the time of paramedic interventions. However, it is expected that any bias would be random across both sexes. It is possible confounders beyond the Utstein variables, such as patient frailty and comorbidities, also influenced paramedic decision making, and these were not controlled for in our study.

## CONCLUSIONS

Our study indicates that, although women received comparable initial resuscitation efforts such as time to pad placement, first defibrillation, and ECC quality to men, women received adrenaline later than men and shorter resuscitation attempts for nonsurvivors. Furthermore, 12‐lead ECGs were obtained more slowly for women, women were less likely to be transported to a tertiary cardiac center, and upon arrival at the hospital they had poorer perfusion status. Considering these disparities, future research should prioritize investigating the underlying causes of these discrepancies, including potential biases in decision making during resuscitation attempts. Furthermore, EMS clinicians should receive targeted education to raise awareness and address implicit biases that may contribute to these disparities, ultimately working toward a more equitable and effective resuscitation care system.

## Sources of Funding

E.N. is supported by a National Health and Medical Research Council Postgraduate Scholarship (number 2003449). Z.N. is supported by a Future Leader Fellowship from the National Heart Foundation of Australia (number 105690).

## Disclosures

None.

## Supporting information

Table S1
