# Supplementary material for: Sex Disparities in Resuscitation Quality Following Out of Hospital Cardiac Arrest
Source: J Am Heart Assoc. 2024 Jun 27;13(13):e033974. doi: 10.1161/JAHA.123.033974 (PMC11255687; doi:10.1161/JAHA.123.033974)
Supplement: Supplementary file 1 — Table S1 [file JAH3-13-e033974-s001.pdf]

SUPPLEMENTAL MATERIAL

Table S1. Sensitivity analysis of resuscitation performance and OHCA outcomes for patients with an attempted resuscitation between March 2019 and June 2023.

		All included patients (n=10,161)	Adjusted for patient age, arrest aetiology, location of OHCA, initial presenting rhythm, witnessed status and presence of bystander CPR.	
			Adjusted odds ratio (95% CI)	Adjusted median difference (95% CI)
**Recognition**	Time (minutes) to place pads, median (IQR)	1 (1 – 2)		0.00 (-0.03 – 0.03)
	Compressions occurring during pad placement, n (%)	4,505 (92.0%)	1.00 (0.79 – 1.26)	
	Correct initial rhythm identification, n (%)	4,657 (93.0%)	1.09 (0.86 – 1.40)	
**ECC quality**	Mean compression rate, median (IQR)	115 (110 - 120)		0.50 (-0.01 – 1.01)
	Mean compression depth, median (IQR)	5.8 (5.1 – 6.5)		0.05 (-0.01 – 0.11)
	Chest compressions fraction, median (IQR)	92 (89 - 93)		0.00 (-0.17 – 0.17)
	Average recoil velocity (mm/s), median (IQR)	379 (337 – 424)		-9.24 (-13.43 – -5.06)
**Defibrillation**	Time (minutes) to first defibrillation, median (IQR)	2 (1 – 3)		0.00 (-0.31 – 0.31)
	Average pre-shock pause (seconds), median (IQR)	5.7 (4.0 – 8.3)		0.08 (-0.24 – 0.40)
	Average post-shock pause (seconds), median (IQR)	3.6 (2.8 – 4.8)		-0.25 (-0.42 – -0.09)
**Advanced interventions**	Time (minutes) to insert supraglottic airway, median (IQR)	3 (2 – 4)		0.00 (-0.12 – 0.12)
	Intubation first pass rate, n (%)	2,748 (83.7%)	1.08 (0.87 – 1.34)	
	Time (minutes) to administer first bolus adrenaline, median (IQR)	7 (4 – 10)		1.00 (0.59 – 1.41)
	Time (minutes) to administer first bolus amiodarone, median (IQR)	2 (1 – 5)		0.00 (-0.64 – 0.64)
**Post-resuscitation care**	Systolic blood pressure ≥ 100mmHg on arrival to hospital, n (%)	1,572 (90.3%)	0.66 (0.47 – 0.93)	
	Time (minutes) to 12-lead ECG acquisition, median (IQR)	6 (4 – 11)		1.00 (0.37 – 1.63)
	Transport to 24-hour PCI facility, n (%)	2.262 (91.3%)	0.69 (0.50 – 0.94)	
	Resuscitation duration for non-survivors (minutes), median (IQR)	20 (5 – 35)		-1.05 (-2.09 – 0.00)

95%CI indicates the 95% confidence interval; ECC, external cardiac compressions; ECG, electrocardiogram; IQR, interquartile range; PCI, percutaneous coronary intervention; OHCA, out-of-hospital cardiac arrest.
